# The Deltoid-Spring Ligament Complex: A Scoping Review and New Segmental Classification

**DOI:** 10.7759/cureus.81715

**Published:** 2025-04-04

**Authors:** Andrey Bilyy, Wai Wai Win Mar, Zain Al Abdeen Al Zuabi, Honey B Frimpong-Manso, Steven Famure, Georgios Solomou, Cecilia Brassett, Chandra Pasapula

**Affiliations:** 1 Plastic and Reconstructive Surgery, Chelsea and Westminster Hospital, London, GBR; 2 Orthopaedics, Queen Elizabeth Hospital King's Lynn, King's Lynn, GBR; 3 Clinical Research, School of Clinical Medicine, University of Cambridge, Cambridge, GBR; 4 Human Anatomy Centre, Department of Physiology, Development and Neuroscience, University of Cambridge, Cambridge, GBR; 5 Trauma and Orthopaedics, Queen Elizabeth Hospital King's Lynn, King's Lynn, GBR

**Keywords:** ankle, ankle and foot, deltoid ligament, deltospring complex, medial collateral ligament, spring ligament

## Abstract

The aim of this study is to review the variation in description of the individual bands comprising the deltoid and spring ligaments in anatomical dissection studies and to propose a novel approach to describe the structure. A literature search for cadaveric studies identifying anatomical variations in the deltoid and spring ligament complexes was conducted using PubMed and Medline databases. The inclusion criteria encompassed human cadaveric dissection studies with measurement of individual deltoid and spring ligament bands in the English language and with full-text availability. The following studies were excluded: animal studies, articles describing surgical repair approaches, and radiological assessment studies without cadaveric dissection. The demographic data, parameters of individual components, as well as the morphological structure of individual deltoid bands were summarised. Out of the 18,208 studies from the database search, 11 articles were included in this study. Thirteen additional studies were obtained from the bibliographies, resulting in a total of 24 studies with 528 ankles evaluated. Due to the complexity of their anatomical relationships, the deltoid and spring ligaments should be described as a single entity: the "deltoid-spring ligament complex". Its gross morphology can be described as triangular, trapezoidal, and rectangular. It can be differentiated into the deep deltoid and the superficial deltospring ligament, which are connected. The latter encompasses the superficial deltoid and superomedial part of the spring ligament. The deep plantar ligament and "the inferior spring ligament" are separate entities reflecting their discrete natures and histological differences. The superficial deltospring ligament can be divided into contiguous segments with variable bands (thickening but not true ligaments). Each segment can be clinically assessed en masse. This description can help to clarify the nomenclature.

## Introduction and background

Ankle sprains are among the most prevalent sports-related injuries, with patients often developing chronic ankle instability characterised by persistent pain and recurrent injuries. As these injuries typically affect the lateral ankle ligament complex, concurrent injuries of the medial ligament complex are often overlooked. Isolated deltoid ligament injuries account for up to 5-15% of all ankle sprains [1], but this may be a significant underestimation. Anatomical variations of the medial ankle ligament complex and the paucity of good clinical tests have contributed to the difficulty in accurate diagnosis and treatment of these injuries [2]. Primary treatment of ankle ligament lesions mainly consists of nonoperative approaches. Surgical interventions may be required to prevent joint degeneration when conservative measures fail and chronic ankle joint instability becomes established, leading to chronic pain and osteoarthritis [3]. Successful operative approaches require a good understanding of the anatomical and biomechanical properties of the medial ankle ligaments.

Current literature identifies the deltoid ligament complex (medial collateral ligament), the spring ligament complex, and the posterior tibial tendon as the key structures that provide medial static and dynamic support to the ankle [4]. Historically, the understanding of the number of layers and individual bands comprising the deltoid ligament has evolved over time, ranging from (a) a single layer with three parts, (b) two layers (superficial and deep), each comprising two distinct ligaments, and to (c) three distinct ligaments in the superficial layer and a single ligament in the deep layer [5]. Currently, the most widely accepted description includes six bands that contribute to the superficial and deep layers of the deltoid ligament. The superficial component comprises the tibiospring, tibionavicular, superficial posterior tibiotalar, and tibiocalcaneal ligaments, while the deep anterior and posterior tibiotalar ligaments constitute the deep layer [6-9]. The tibiospring, tibionavicular, and the deep posterior tibiotalar portions are thought to be always present, whereas the presence of the superficial posterior tibiotalar, tibiocalcaneal, and deep anterior tibiotalar components may vary in individuals [10]. Anatomically, the deltoid ligament spans the tibiotalar, subtalar, and talonavicular joints. Functionally, the superficial deltoid layer prevents eversion of the hindfoot while the deep deltoid component restricts external rotation of the talus, thereby stabilising the medial ankle and limiting anterior, posterior, and lateral translation of the talus and restraining talar abduction at the talocrural joint [7,11].

Similarly, descriptions of the spring ligament complex have been inconsistent and created confusion in the literature [4,6]. The currently accepted description of the spring ligament complex (also known as the plantar calcaneonavicular ligament) involves two components: the superomedial calcaneonavicular and inferior calcaneonavicular ligaments. The inferior calcaneonavicular ligament is thought to be multifascicular and is further subdivided into medioplantar oblique and inferoplantar longitudinal bands [6]. The notion that the individual components of the complex are distinct structures is a major contributor to the confusion [6]. For example, as the superomedial calcaneonavicular ligament is directly attached to the superficial deltoid ligament as well as to the medioplantar oblique portion of the inferior calcaneonavicular ligament, the components cannot be easily separated during anatomical dissections [6]. Functionally, both the spring ligament complex and the deltoid ligament complex act together to stabilise the medial ankle joint.

This study aims to review available literature on anatomical dissections of the deltoid and spring ligament complexes, evaluate the variation in terminology and numbers of components and report on the documented nomenclature of these bands. Finally, the authors propose a novel, segmental approach to anatomical description of the deltoid and spring ligament complexes that takes into account their interconnectedness and eliminates potential difficulties of identifying individual components.

Methods

A literature search was conducted using PubMed and Medline databases, including all articles up to June 2023. A list of anatomy- and ligament-related Medical Subject Headings (MeSH) terms was generated. The anatomy-related terms included difference, variation, variability, anatomical, anatomic, anatomy, morphology, morphologic, morphological, structure, location, configuration, arrangement, foot, and ankle and were combined using the Boolean term “OR”. The list of ligament-related search terms was comprised of spring ligament, plantar calcaneonavicular ligament, superomedial calcaneonavicular ligament, medioplantar oblique ligament, inferior calcaneonavicular ligament, medial collateral ligament, articular ligaments, deltoid ligament, medial ankle collateral, tibiocalcaneal, tibiospring, and tibionavicular ligament, which were then combined using “OR”. The articles produced within each category were then combined using the Boolean term “AND” to restrict the search output. The final output was then limited to the English language and articles with full text availability. The references from the generated articles were further evaluated for additional studies.

All papers were uploaded onto Rayyan software (Rayyan, Cambridge, MA), where deduplication and abstract and full-text screening were performed by the authors. Any disputes were resolved by the expert in the field (CP), an orthopaedic foot and ankle surgeon with over 15 years of experience. The inclusion criteria encompassed human cadaver dissection studies visualising and measuring individual deltoid and spring ligament bands in the English language with full text. Descriptive radiological studies that included anatomical dissection of the ligaments prior to imaging were included in this review. Studies were excluded based on the following criteria: animal studies, articles describing surgical repair approaches, and radiological assessment studies without prior anatomical dissection. The demographic data, ligament parameters, individual bands of the deltoid and spring ligaments, as well as the morphological structure of individual deltoid bands identified in the selected studies were summarised.

## Review

Results

**Figure 1:**
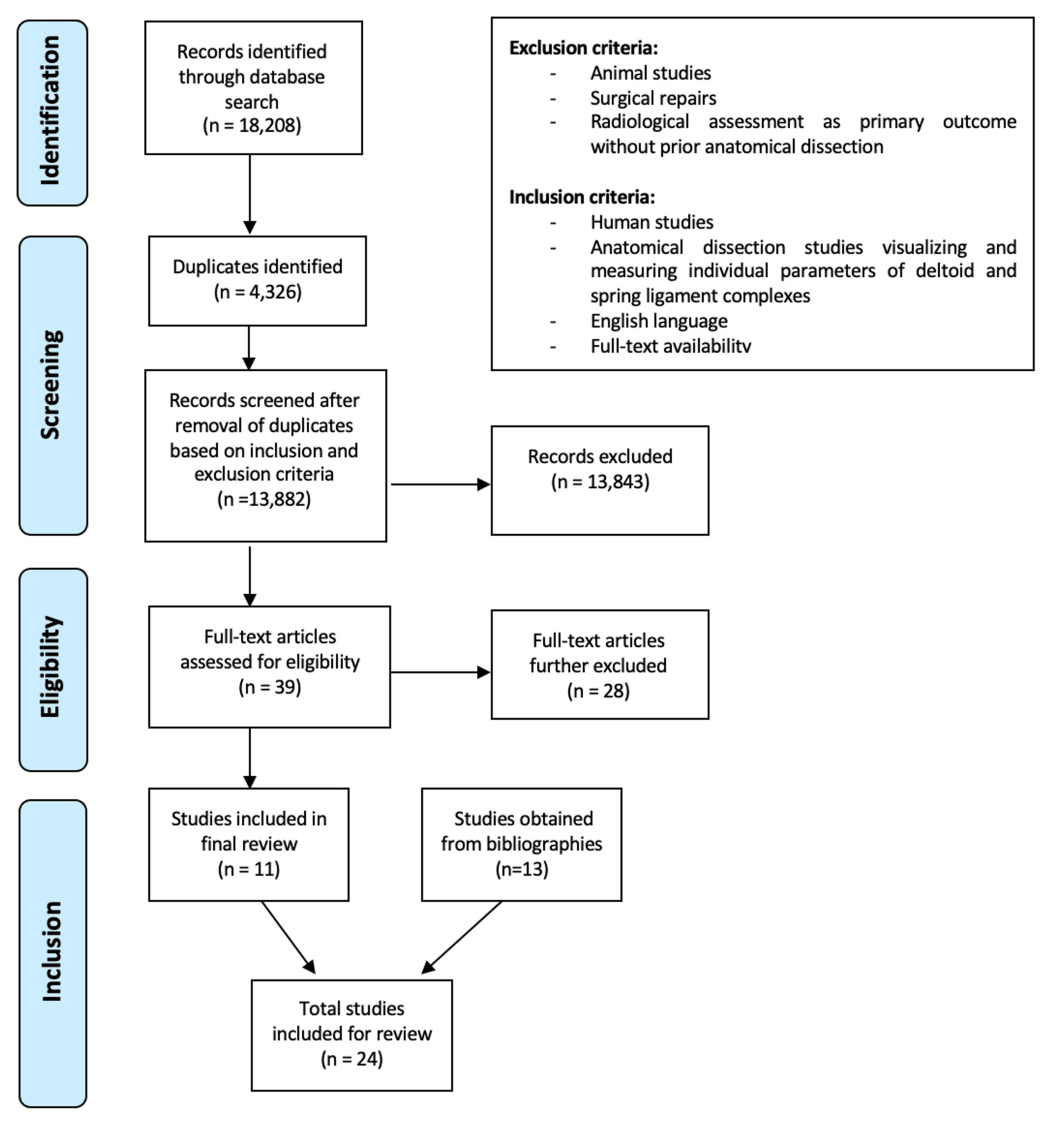
Literature search summary.

The database search generated 18,208 results. After identification, 4326 duplicates were removed, thereby leaving 13,882 studies for abstract screening. Based on the inclusion and exclusion criteria, 13,843 records were excluded, and 39 studies underwent full-text evaluation. Full-text screening resulted in the exclusion of a further 28 studies, generating 11 articles for in-depth review. Thirteen additional studies were obtained from bibliographies, resulting in a total of 24 included in this report (Figure 1).

The 24 studies evaluated a total of 528 cadaveric ankles, although some articles did not mention the exact number of specimens analysed in their studies [10,12]. It is also difficult to comment on whether there is any difference in anatomy between the left and right sides, as well as between sexes, since significance was not reported in detail. However, more right-sided ankles were reported together with more female cadavers. The donors’ mean age was in the range of 50.4-83 years (Table 1).

**Table 1:** Summary of included studies. * Studies used non-paired cadaveric specimens; hence, the number of ankles is equal to the number of cadavers. ** Two separate studies were included in the report, but only one is tabulated herein due to the same subject pool being used by the authors. *** The study included was a review and had no sample size mentioned. “-” Information not available.

Study (authors, publication, country)	Mean age (years)	No. of cadavers	No. of ankles	Laterality		Sex		Mean height (m)	Mean weight (kg)	Average foot length (cm)
				Right	Left	Male	Female			
Clanton et al. (2015), USA [1]	56.00	12*	12*	-	-	7	5	-	-	-
Siegler et al. (1988), USA [3]	67.8	20*	20*	-	-	8	12	1.71	69.10	-
Pankovich et al. (1979), USA [5]	-	16*	16*	-	-	-	-	-	-	-
Cromeens et al. (2015), USA [6]	73.00	9*	9*	-	-	6	3	-	-	-
Milner et al. (1998), UK [8,9] **	83.00	20	40	-	-	8	12	-	-	-
Golano et al. (2010), Spain [10] ***	-	-	-	-	-	-	-	-	-	-
Campbell et al. (2014), USA [11]	50.40	14*	14*	6	8	8	6	-	-	25.20
Savage-Elliott et al. (2012), USA/Spain [12] ***	-	-	-	-	-	-	-	-	-	-
Panchani et al. (2014), USA [13]	76.60	17	33	17	16	8	9	-	-	-
Zamperetti et al. (2018), Italy/Spain [14]	75.87	15*	15*	8	7	6	9	-	-	23.00
Apoorva et al. (2014), India [15]	-	60*	60*	-	-	-	-	-	-	-
Babacan et al. (2022), Turkey [16]	-	15	30	-	-	13	2	49.56	--	23.42
Wenny et al. (2015), Japan [17]	-	16*	16*	-	-	-	-	-	-	-
Luo et al. (1997), USA [18]	61.40	11*	11*	6	5	5	6	-	-	-
Guerra-Pinto et al. (2021), Portugal/Spain [19]	78.60	40	43	-	-	19	24	-	-	-
Boss et al. (2002), Switzerland [20]	75.50	12*	12*	6	6	-	-	-	-	-
Ismail et al. (2022), SAU [21]	75.00	30*	30*	18	12	16	14	-	-	-
Won et al. (2016), S. Korea [22]	64.70	39	60	-	-	22	17	-	-	-
Mengiardi et al. (2005), Switzerland [23]	76.00	5*	5*	3	2	1	4	-	-	-
Taniguchi et al. (2003), Japan [24]	-	-	48	-	-	-	-	-	-	-
Patil et al. (2007), USA [25]	74.00	15	30	-	-	5	10	-	-	-
Davis et al. (1996), USA [26]	-	14*	14*	-	-	-	-	-	-	-
Schneck et al. (1992), UK [27]	-	10*	10*	-	-	-	-	-	-	-

Components of the deltoid ligament

Constant Superficial Bands

Tibiospring ligament: It is the most superficial band that extends distally and almost perpendicularly from its origin on the medial malleolus to form the most superficial attachment. The distal insertion is the superior border of the spring ligament and the plantar calcaneonavicular ligament. The average width is 9.0 mm while the mean length and mean thickness are 18.5-24.3 mm and 1.50 mm, respectively (Tables 2, 3).

**Table 2:** Parameters of the individual bands of the deltoid and spring ligaments across all studies. * Range values provided only where available. Px: proximal; Dx: distal; ATTL: anterior tibio-talar ligament; dPTTL: deep posterior tibio-talar ligament; TTC: tibio-talo-calcaneal; TCL: tibio-calcaneal ligament; TNL: tibio-navicular ligament; SMCN: superior medial calcaneo-navicular; n/a: information not available.

Ligament	Ligament components	Ligament band identified (%)	Mean length, range* (mm)	Mean width, range* (mm)	Mean thickness, range* (mm)	Origins	Insertions	Citation
Deltoid ligament	Tibiotalocalcaneal	100.00	n/a	n/a	n/a	Antero-medial medial malleolus	Superomedial aspect of Sustentaculum tali + talus	[21]
	Anterior tibiotalonavicular	60.00	n/a	n/a	n/a	Distal antero-lateral medial malleolus	Anterior navicular bone	[21]
	Tibiocalcaneonavicular	100.00	23.20-33.51	Px: 17.98; Dx: 40.88	3.70	Medial malleolus (anteriorly to navicular + inferiorly to calcaneus)	Delta-shaped attachment to the anteromedial surface of the anterior colliculus of the tibial malleolus	[6,14]
	Tibionavicular	89.00-100.00	27.80-37.20	Px: 11.00; Dx: 27.50	n/a	Anterior border of medial malleolus	Dorsomedial of navicular	[1,3,5,8-13,15,18,19,22,27]
	Tibiocalcaneal	15.00-100.00	10.30-30.10	Px: 9.50; Dx: 22.00	0.30-3.30	Medially at the anterior colliculus	Medial border of sustentaculum tali	[1,3,5,8-13,15,18-20,22,27]
	Intermediate tibiotalar	30.00	n/a	n/a	n/a	Anteromedial medial malleolus, medial to ATTL connection + under TTC fasciculus	Anterior edge of sulcus tali	[21]
	Deep anterior tibiotalar	40.00-86.00	n/a	n/a	n/a	Anterolateral medial malleolus	Anteromedial talus	[1,5,6,8-15,17-22,27]
	Deep posterior tibiotalar	n/a	11.86	n/a	45.2	n/a	n/a	[1,3,5,6,8-15,17-22,27]
	Tibiospring	100.00	18.50-24.30	9.00	1.50	(1) Anterotibial colliculus OR (2) medial malleolus	Superior border of spring ligament (plantar calcaneo-navicular)	[1,3,8-12,15,19,20,22,27]
	Fibres to spring	46.00	6.00	n/a	n/a	Between TCL + TNL proximal attachments	Superior spring ligament	[13]
	Superficial talotibial	93.75	14.00-30.40	n/a	n/a	Postero-medial surface of the anterior colliculus + adjacent small part of the posterior colliculus	Medial tubercle of talus + sustentaculum tali	[5]
	Superficial posterior tibiotalar	37.50-97.00	11.64-24.30	8.00	0.90-1.27	Postero-medial aspect of medial malleolus + posterior colliculus	Medial talar tubercle + sustentaculum tali	[1,6,8-15,19-22]
	Deep anterior tibiotalar	10.00-93.00	11.50-19.60	Px: 7.50; Dx: 7.70	0.96-1.20	Anterior colliculus + intercollicular groove of medial malleolus	Medial talus distal to antero-medial articular facet	[1,5,6,8-15,17-22,27]
	Deep posterior tibiotalar	90.00-100.00	9.50-23.60	Px: 12.49; Dx: 15.54	0.60-1.60	(1) Intercollicular groove OR (2) posterior surface of anterior colliculus OR (3) anterior surface of posterior colliculus	Medial talus under tail of articular facet to posteromedial talar tubercle	[1,3,5,6,8-15,17-22,27]
	Deep intermediate tibiotalar	20.00	12.67	Px: 7.90; Dx: 8.39	1.22	Posterior intercollicular groove	Posterior colliculus & anterior facies malleolaris medialis	[14]
	Deep to tibiocalcaneal	12.00	12.67	Px: 7.90; Dx: 8.39	1.22	Between ATTL + dPTTL	Supero-medial aspect of talus between ATTL & dPTTL	[13]
	Posterior to sustentaculum tali	6.00	n/a	n/a	n/a	Superomedial sustentaculum tali + anterior calcaneus	Medial surface of calcaneus	[13]
Spring ligament	Superomedial calcaneonavicular	100.00	n/a	n/a	17.00-34.60	Superomedial calcaneus	Navicular facet	[20,23-26]
	Inferoplantar longitudinal	100.00	15.90	5.20	1.70	Inferior calcaneus stretched anteromedially	Navicular beak	[6,23-25]
	Inferior calcaneonavicular	n/a	6.00	n/a	n/a	Coronoid cavity (notch between middle & anterior calcaneus)	Anterior mid-navicular immediately lateral to SMCN	[20,26]
	Medioplantar oblique	n/a	n/a	n/a	n/a	Coronoid fossa of calcaneus	Medioplantar portion of navicular bone	[23-25]
	Inferior calcaneonavicular	n/a	4.00-9.00	n/a	1.00-3.30	Notch between the middle and anterior calcaneal facets (coronoid cavity)	Inferior surface of midnavicular	[20,26]

**Table 3:** Superficial and deep bands of the deltoid ligament identified in the included studies. * Same subject pool used for both studies and published by the same authors in the same year. TCN: tibio-calcaneonavicular ligament; PTT: posterior tibiotalar; ATT: anterior tibiotalar; TT: tibiotalar; Ant: anterior.

Study	Superficial					Deep			
Clanton et al. (2015), USA [1]	Tibiocalcaneal	Tibionavicular	Tibiospring	Superficial PTT		Deep PTT	Deep ATT		
Siegler et al. (1988), USA [3]	Tibiocalcaneal	Tibionavicular	Tibiospring			Deep PTT			
Pankovich et al. (1979), USA [5]	Tibiocalcaneal	Tibionavicular		Superficial talotibial		Deep PTT	Deep ATT		
Cromeens et al. (2015), USA [6]	TCN			Superficial PTT		Deep PTT	Deep ATT	Inferoplantar longitudinal	
Milner et al. (1998), UK [8] *	Tibiocalcaneal	Tibionavicular	Tibiospring	Superficial PTT		Deep PTT	Deep ATT		
Milner et al. (1998), UK [9] *	Tibiocalcaneal	Tibionavicular	Tibiospring	Superficial PTT		Deep PTT	Deep ATT		
Golano et al. (2010), Spain [10]	Tibiocalcaneal	Tibionavicular	Tibiospring	Superficial PTT		Deep PTT	Deep ATT		
Campbell et al. (2014), USA [11]	Tibiocalcaneal	Tibionavicular	Tibiospring	Superficial PTT		Deep PTT	Deep ATT		
Savage-Elliott et al. (2012), USA/Spain [12]	Tibiocalcaneal	Tibionavicular	Tibiospring	Superficial PTT		Deep PTT	Deep ATT		
Panchani et al. (2014), USA [13]	Tibiocalcaneal	Tibionavicular	Fibres to spring	Superficial PTT	Band posterior to Sustentaculum tali	Deep PTT	Deep ATT	Deep to tibiocalcaneal ligament	
Zamperetti et al. (2018), Italy/Spain [14]	TCN			Superficial PTT		Deep PTT	Deep ATT	Deep intermediate TT	
Apoorva et al. (2014), India [15]	Tibiocalcaneal	Tibionavicular	Tibiospring	Superficial PTT		Deep PTT	Deep ATT		
Wenny et al. (2015), Japan [17]	Deltoid					Deep PTT	Deep ATT		
Lou et al. (1997), USA [18]	Tibiocalcaneal	Tibionavicular				Deep PTT	Deep ATT		
Guerra-Pinto et al. (2021), Portugal/Spain [19]	Tibiocalcaneal	Tibionavicular	Tibiospring	Superficial PTT		Deep PTT	Deep ATT		
Boss et al. (2002), Switzerland [20]	Tibiocalcaneal		Tibiospring	Superficial PTT		Deep PTT	Deep ATT		
Ismail et al. (2022), SAU [21]	Tibiotalocalcaneal			Superficial PTT		Deep PTT	Deep ATT	Intermediate TT	Ant: Tibio-talo- navicular
Won et al. (2016), S. Korea [22]	Tibiocalcaneal	Tibionavicular	Tibiospring	Superficial PTT		Deep PTT	Deep ATT		
Schneck et al. (1992), UK [27]	Tibiocalcaneal	Tibionavicular	Tibiospring			Deep PTT	Deep ATT		

Tibionavicular ligament: It is the most anterior band that originates from the anterior border of the anterior colliculus of the medial malleolus and extends in a fan-shaped fashion, forming a triangular ligament. It is attached distally to the dorsomedial surface of the navicular, with occasional fibres extending into the spring ligament. Its reported mean length is 27.8-37.2 mm, with the average width at the proximal, middle, and distal attachments being 11.0 mm, 13.5 mm, and 27.5 mm, respectively. Its tibial footprint is 16.1 mm, and the navicular part is 9.7 mm (Tables 2, 3).

Tibiocalcaneal ligament: It is the strongest band, taking its origin from the medial surface of the anterior colliculus and attaching distally to the medial border of the sustentaculum tali of the calcaneus, with a few fibres occasionally inserting into the spring ligament. Its average length is 10.3-30.1 mm, with the thickness ranging from 0.3 to 3.3 mm. The width is 9.5 ± 3.9 mm proximally, 12.0 ± 5.8 mm in the middle part, and 22.0 ± 14.3 mm distally (Table 2).

Superficial posterior tibiotalar ligament: Despite some slight differences in descriptions of its origin and insertion between studies, this is described as originating from the medial surface of the posterior colliculus and the posterior part of the anterior colliculus, distal to both the medial talar tubercle and sustentaculum tali. The reported length ranges from 11.64 to 24.3 mm with a width of 8.0 ± 2.8 mm. The thickness is between 0.9 and 1.27 mm (Tables 2, 3).

Additional Superficial Variants

Band posterior to sustentaculum tali: Panchani et al. (2014) [13] reported an additional superficial variant found in two specimens (2/33, 6%), which was termed a band posterior to sustentaculum tali. The band shared a proximal attachment with the tibiocalcaneal ligament and coursed distally, posterior to the sustentaculum tali on the medial surface of the calcaneus (Tables 2, 3).

Constant Deep Bands

Deep posterior tibiotalar ligament: It is the strongest and thickest of the deep ligaments. It passes from the intercollicular groove and the posterior surface of the anterior colliculus to insert onto the medial surface of the talus under the tail of the articular facet as far as the posteromedial talar tubercle. The length ranges between 9.5 and 23.6 mm on average, with its thickness being between 0.6 and 1.6 mm. Its proximal and distal widths are 12.49 mm and 15.54 mm, respectively (Tables 2, 3).

Deep anterior tibiotalar: The deep anterior tibiotalar ligament originates from the anterior colliculus and intercollicular groove of the medial malleolus and is attached distally to the medial surface of the talus just distal to the anterior part of the medial articular facet and deep to the posterior tibiotalar ligament insertion. Its length and thickness range from 11.5 to 19.6 mm and 0.96 to 1.20 mm, respectively. The average proximal width is 7.5 mm, with the distal width being 7.70 mm (Tables 2, 3).

Additional Deep Variants

Band deep to tibiocalcaneal ligament: An additional band of the deep layer reported by Panchani et al. (2014) [13] was identified between the deep anterior and posterior tibiotalar ligaments in four of 33 ankles (12%). Zamperetti et al. (2018) [14] also identified this band in three of 15 ankles (20%) and called it the deep intermediate tibiotalar ligament, originating between the deep anterior and posterior tibiotalar ligaments and attaching distally to the superomedial aspect of the talus between these two ligaments. The length and thickness were reported as 12.67 ± 2.08 mm and 1.22 ± 0.36 mm, respectively. Its proximal insertion area is 7.9 ± 0.46 mm, and the distal insertion area is 8.39 ± 0.58 mm (Tables 2, 3).

Components of the spring ligament

The spring ligament, also known as the plantar calcaneonavicular ligament (CNL), extends from the sustentaculum tali of the calcaneus to the plantar aspect of the navicular bone. It consists of two components: the superomedial and inferior calcaneonavicular ligaments. The inferior calcaneonavicular ligament is further divided into two fascicles: medioplantar oblique CNL and inferoplantar longitudinal CNL. The superomedial CNL courses from the medial aspect of the sustentaculum tali, bypassing the tuberosity of the navicular and attaching distally onto the superomedial aspect of the navicular. The medioplantar oblique CNL extends from a region just anterior to the middle articular facet of the calcaneus in the coronoid fossa to the medioplantar portion of the navicular, while the inferoplantar longitudinal CNL attaches from the coronoid fossa of the calcaneus anterior to the medioplantar oblique CNL to the inferior beak of the navicular (Tables 2, 4).

**Table 4:** Spring ligament bands identified in respective studies.

Study	Bands identified
Boss et al. (2002), Switzerland [20]	Superomedial calcaneonavicular, inferior calcaneonavicular
Mengiardi et al. (2005), Switzerland [23]	Superomedial calcaneonavicular, medioplantar oblique calcaneonavicular, inferoplantar longitudinal calcaneonavicular
Taniguchi et al. (2003), Japan [24]	Superomedial calcaneonavicular, medioplantar oblique calcaneonavicular, inferoplantar longitudinal calcaneonavicular
Patil et al. (2007), USA [25]	Superomedial calcaneonavicular, medioplantar oblique calcaneonavicular, inferoplantar longitudinal calcaneonavicular
Davis et al. (1996), USA [26]	Superomedial calcaneonavicular, inferior calcaneonavicular

Discussion

Systematic reviews evaluating the anatomy of the deltoid-spring ligament (DSL) complex are lacking. While DSL descriptions are varied and lacking in uniformity, it is implicated in the pathogenesis of several foot conditions.

**Figure 2:**
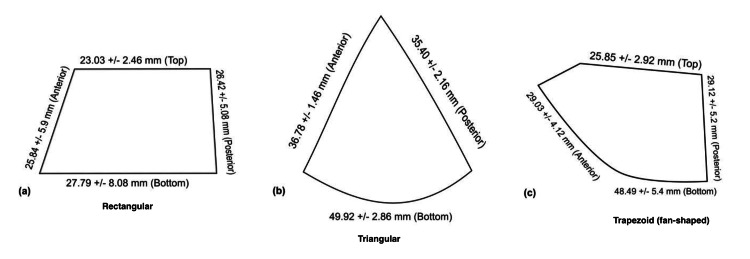
Variations in morphological appearance of the superficial layer of the deltoid ligament. References [15,16]. Image credit: Zain Al Abdeen Al Zuabi.

The structure can be considered on multiple levels. In terms of gross morphology (Table 5), Apoorva et al. (2014) [15] evaluated the superficial layer of the DSL in 60 cadaveric ankles and described three shapes: 72% trapezoidal (n = 43), 20% rectangular (n = 12), and 8% triangular (n = 5) (Figure 2). Babacan et al. (2022) [16] identified two types in their study: 24 trapezoidal (wide inferior portion) (80%) and six rectangular (20%). The trapezoidal variants were almost twice as long at the base compared to the rectangular ones. The smaller dimensions reflect less tissue, which may reflect susceptibility to clinical ligament failure.

**Table 5:** Gross morphology of the deltoid ligament. “-” Shape not identified.

Study	Morphological shape		
	Trapezoidal	Rectangular	Triangular
Apoorva et al. (2014) [15]	43/60 (72%)	12/60 (20%)	5/60 (8%)
Babacan et al. (2022) [16]	24/30 (80%)	6/30 (20%)	-

Wenny et al. (2015) [17] analysed DSL gross lengths and tibial attachment areas. The mean tibial area of attachment was 1.13 ± 0.15 cm^2^, with a centre of insertion located about 9.5 mm from the tip of the medial malleolus and 6.3 mm from its anterior border. This places the attachment in the area commonly related to medial malleolus fractures. The true disruption of the capsule may, therefore, be far greater than that previously recognised. The distal attachment connects to three structures and has a greater area. The dorsal lateral navicular surface attachment was 1.19 ± 0.12 cm^2^, and the calcaneal attachment at the sustentaculum tali was 1.13 ± 0.24 cm^2^. The smaller insertion area proximally may help to localise stress within the ligament. The differential natural strain in parts of the same ligament may afford greater protection from injury for some ligaments compared to other areas. The distal area may have less potential for disruption.

Due to its non-homogeneous nature, the deltoid ligament shows different straining patterns depending on the joint through which it acts. Luo et al. (1997) [18] evaluated 11 cadaveric feet and ankle specimens using a mounted jig. The largest ligament was the tibionavicular ligament by length (3.25 ± 0.47 cm), which had a large elongation (0.5-1 cm) in plantarflexion as a result of the large rotation of 29.5° at the ankle joint. The tibiocalcaneal ligament elongation was large and maximal in dorsiflexion (0.26 ± 0.11 cm). Avulsion fractures at sites of insertion may imply the presence of DSL disruption. Gregersen et al. (2022) [2] have also reported differential straining of individual deltoid bands. They found that the tibiospring ligament is maximally tense in plantarflexion, while the tibiocalcaneal and deep posterior tibiotalar ligaments are most tense (thus providing stability) in dorsiflexion. The authors concluded that valgus/varus and rotational movements primarily affected the length and tensile strength of the superficial layer as well as the deep anterior tibiotalar ligament, but not the deep posterior tibiotalar band. Similarly, Takao et al. (2020) [7] evaluated the straining patterns of the deltoid superficial layer. They found that the tibionavicular, tibiospring, tibiocalcaneal, and superficial posterior tibiotalar ligaments worked most efficiently (providing the greatest tensile strength) in plantarflexion-abduction, abduction, dorsiflexion-abduction (pronation), and dorsiflexion, respectively. In a clinical setting, this information can be used to evaluate the ligament complex by assessing the range of pain across different ankle movements.

Concept of one ligament

Due to their complex interconnections, the deltoid and spring ligament complexes may be better interpreted as one single complex (the DSL complex). Amaha et al. (2019) [28] presented their findings from 22 cadaveric joint capsules of the ankle, subtalar, and talonavicular joints from anterior to posteromedial as one continuous sheet detached from the sustentaculum tali of the calcaneus. This single continuous joint capsular structure, comprising the deltoid-spring complex, with fibrous and synovial connective tissues forming a sheath floor for the tibialis posterior tendon, further demonstrates the single-structure nature of the ligament. The ligament is not uniform histologically, reflecting the demands relevant to different areas. The authors described the deep deltoid layer as being the fibrous portion of the continuous capsule, rather than as a separate structure. Additionally, the capsule covering the medial part of the subtalar and talonavicular joints comprises cartilaginous tissue, which was previously known as the superomedial portion of the spring ligament. Furthermore, the anterior capsule is mainly collagenous, while the anteromedial capsule is fibrous with a cartilaginous covering of the talus.

This interconnectedness of different elements of the DSL highlights the problems with describing the ligament in terms of discrete bands. Band descriptions in the literature vary in nomenclature, anatomy, and numbers identified (Table 3). The lack of concordance may reflect variations in dissection technique through artificial divisions of the fascicles/bands during the process [28]. The most widely accepted descriptions to date recognise six bands [1,8-12,15,19], although some describe eight bands [13] with variable presence of the superficial and deep components (Table 3). Other interpretations with five bands comprising the DSL complex also exist [5,6,20], and Cromeens et al. (2015) [6] acknowledged the challenge in dissecting the different ligaments; thus, true differentiation of the ligaments may not be possible. In contrast to individual band separation, the authors of the present study propose that the superficial layer of the DSL may be better classified in terms of segmental contiguous areas of variable thickening (anterior, middle, and posterior) rather than as discrete bands.

Anterior Segment

Pankovich et al. (1979) [5] and Panchani et al. (2014) [13] identified the whole anterior structure as one large tibionavicular ligament originating from the anterior colliculus as a fan-shaped triangular ligament inserting into the dorsomedial navicular surface and the dorsomedial surface of the spring (calcaneonavicular) ligament. Pankovich et al. (1979) [5] did not consider the fibres attaching to the spring ligament as a separate ligamentous band. The authors described the tibionavicular fascicle as the largest, widest, yet weakest portion of the superficial deltoid, blending with the joint capsule anterolaterally and with the tibiocalcaneal ligament posteriorly. Gregersen et al. (2022) [2] did not describe the talonavicular ligament as a separate ligament, as they could not separate this from the capsule, consistent with Boss et al. (2002) [20].

Cromeens et al. (2015) [6] incorporated this concept of the tibiocalcaneal ligament to describe the spring ligament and all ligamentous structures attaching to the navicular as the tibiocalcaneonavicular ligament. According to the authors, the tibiocalcaneonavicular ligament includes structures from both the deltoid ligament (the tibiocalcaneal, tibionavicular, and tibiospring bands) as well as spring ligament complexes (the superomedial calcaneonavicular and medioplantar oblique bands), further indicating the unity of the structure. Variations in individual ligament/band attachments have been described in this anterior segment. Milner et al. (1998) [8,9] found significant band variations in 40 cadaveric ankles. The constant bands were the tibiospring, tibionavicular, and deep posterior tibiotalar ligaments. We agree with Pankovich et al. (1979) [5] and Panchani et al. (2014) [13] in their description that all the structures associated with the navicular should form part of an anterior segment that helps resist anterior draw and talonavicular abduction.

Middle Segment and Tibiotalocalcaneal Ligament

Pankovich et al. (1979) [5] described the tibiocalcaneal ligament as part of a middle band structure, originating from the mid-medial anterior colliculus surface to insert along the medial sustentaculum tali border of the calcaneus. Panchani et al. (2014) [13] identified the tibiocalcaneal ligament in 94% of ankles (31 of 33). Band variation occurred in the middle segment, with Panchani et al. (2014) [13] identifying an additional variant in the superficial layer, which they termed a band posterior to sustentaculum tali. Additionally, the researchers identified three deep ligaments in total - the commonly accepted deep anterior and posterior tibiotalar bands separated by an additional fascicle, which they called a band deep to the tibiocalcaneal ligament (Table 3). The deep anterior tibiotalar ligament was found in 86% (30 of 33) of the specimens, while the deep posterior tibiotalar ligament was identified in 100% of the subjects (33 of 33) and was the thickest and widest band.

Guerra-Pinto et al. (2021) [19] identified the tibiocalcaneal ligament in 77% (33). In the absence of this band (23%), all cases (10) had some fibres spanning the gap between the tibiospring ligament and the sustentaculum tali. Several authors believe this ligament is part of the tibiospring ligament. For example, Panchani et al. (2014) [13] reported that the tibiocalcaneal fibres were so interconnected with the fibres to the tibiospring ligament that the authors were only able to isolate them clearly in 15 out of 33 ankles (46%). Furthermore, Pankovich and Shivaram [5] did not report the tibiospring as a separate band at all, as they believed that the tibiospring fibres were part of the tibionavicular band.

**Figure 3:**
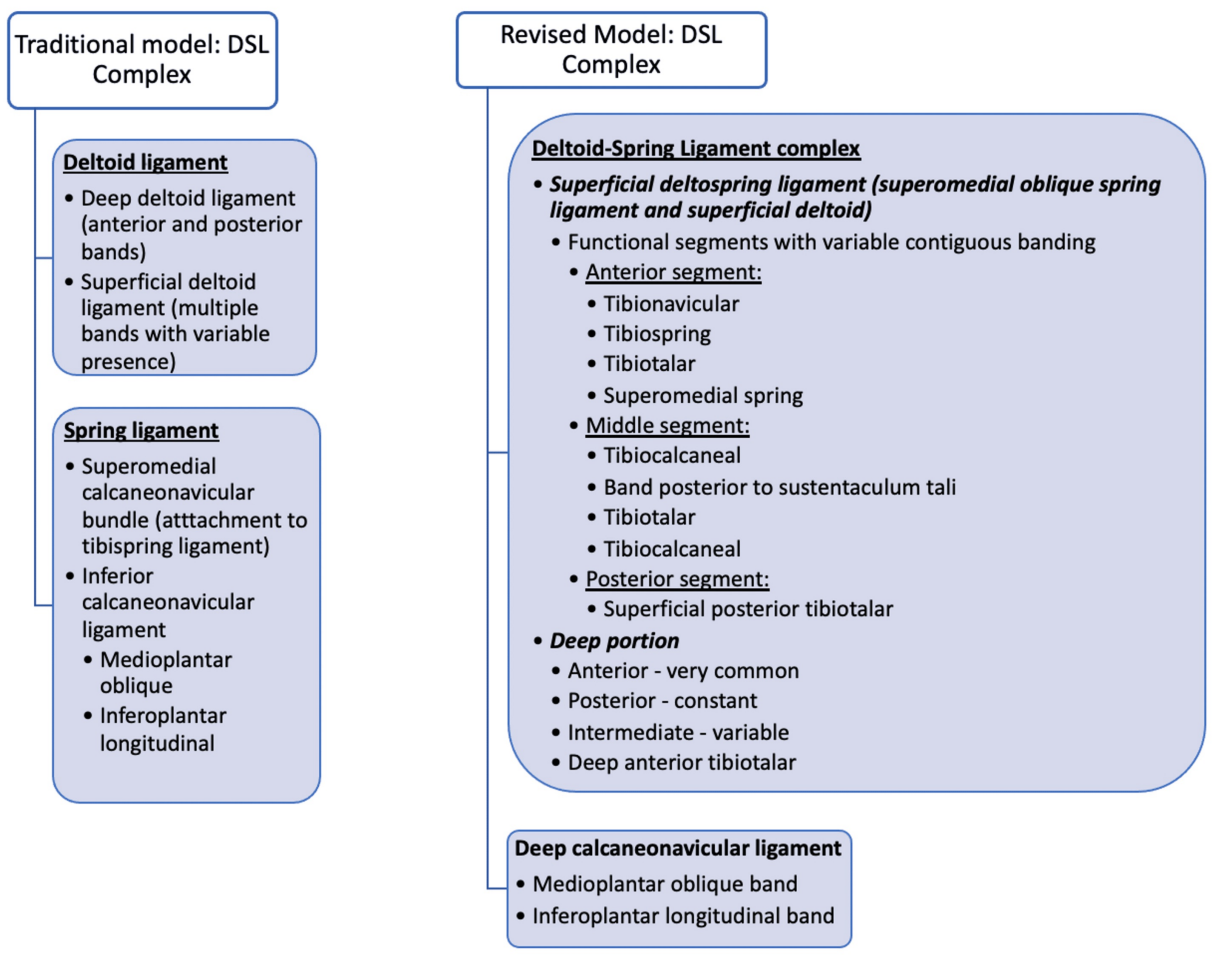
Traditional vs. revised model of the deltoid-spring ligament (DSL) complex. The revised model considers the superomedial portion of the spring ligament as part of the superficial deltoid. This combined ligament is referred to as the superficial deltospring ligament. The inferior spring ligament has been renamed as the deep calcaneonavicular ligament. Terms such as "plantar spring" and "inferior spring" detract from anatomical and functional understanding and imply the superomedial spring ligament portion is separate/distinct from the superficial deltoid, placing it closer to the deep calcaneonavicular ligament to suggest this is one structure/complex. This revised structure and terminology help aid understanding. Image credit: Andrey Bilyy, Zain Al Abdeen Al Zuabi, and Chandra Pasapula.

Ismail et al. (2022) [21] identified a tibiotalocalcaneal ligament (TTC) in 30 specimens (100%) as a part of the superficial layer, spanning from the anteromedial side of the medial malleolus to the superomedial aspect of the sustentaculum tali. Within the isolated tibiotalocalcaneal bands, further variations were identified. The researchers found TTC variants with a complex talar attachment whereby the posterior fascicles extended to the medial edge of the sulcus tali (n = 6, 20%). Additionally, in six ankles (20%), double-banded TTCs were isolated, signifying further complexity of the middle segment of the DSL. Interestingly, the authors were able to identify four total fascicular bundles in the deep layer as opposed to the conventionally accepted two: an intermediate tibiotalar ligament between the anterior tibiotalar and superficial posterior tibiotalar ligaments in nine specimens (30%), a deep posterior tibiotalar (27 of 30, 90%), an anterior tibiotalonavicular (18 of 30, 60%) and an anterior tibiotalar ligament (12 of 30, 40%). Such variation in the deep layer may be attributable to the dissection approach used by the researchers. In our model (Figure 3), we recognise all the fibres that originate from the tibia anterior colliculus and insert in the direction of/onto the sustentaculum tali fragment as part of the middle segment fibres. Superficial deltoid fibres that also insert onto the talus in this region as part of the TTC ligament can also be considered middle segment fibres. The middle segment is also functionally different to the anterior segment when considering surgical reconstruction.

Posterior Segment

Several authors [5,13,19,21] have described the superficial posterior tibiotalar ligament as a variably present, posterior-most bundle of the deltoid ligament that originates from the posteromedial surface of the anterior colliculus and takes a posterodistal course to insert into the anterior portion of the medial talar tubercle. Its inconstant presence has been previously corroborated in the literature [1,5,8,9,11,13,19,20]. Cromeens et al. (2015) [6] described the ligament as being present in all nine ankles (100%) in their report, but its attachment to the calcaneus, tibia, and talus varied between specimens. In contrast, Gregersen et al. (2022) [2] could not identify the superficial posterior tibiotalar band in their study of 10 cadaveric ankles, despite the identification by other authors. The reported prevalence of the band has ranged widely from 37.5% to 79% in other studies [1,8,9,11,20], depending on the dissection method used. Won et al. (2016) [22] reported the prevalence rate of the superficial posterior tibiotalar ligament in their study as 83.3% (50/60). In our model, we attribute all superficial fibres that originate on the posterior colliculi of the medial malleolus and attach posterior to the sustentaculum tali fragment as part of the posterior segment.

The spring ligament (SL) complex

The SL complex is not a single structure and comprises traditionally two ligaments (superomedial and inferior calcaneonavicular), whose primary function is to stabilise the medial longitudinal arch and head of the talus. The superomedial calcaneonavicular bundle originates from the medial and anterior border of the sustentaculum tali to insert on the superomedial aspect of the navicular. It is connected to the tibiospring, tibionavicular, and tibiocalcaneal bands of the superficial deltoid layer, thereby reinforcing the intimate functional connection between the "spring ligament" and "deltoid ligament" complexes [4,20]. The inferior calcaneonavicular ligament is multifascicular and originates in the coronoid fossa of the calcaneus to insert onto the navicular beak/tuberosity. It is recognised to have two fascicular contributions referred to as the medioplantar oblique and inferoplantar longitudinal ligaments [23,24] (Table 4). Similar to the deltoid ligament complex, individual bands of the spring ligament are difficult to separate consistently, with some reports stating that the medioplantar oblique fibres may not always be clearly dissected from those of the superomedial component [23,25]. The medioplantar oblique and inferoplantar longitudinal ligaments have a more tensile-load function than the superomedial band [26]. Histological analysis has shown that the spring ligament complex primarily consists of densely packed collagen fibres without any elastic properties, and therefore, the word spring is now considered a misnomer. The complex is believed to provide more of a sling function for the talar head [25,26].

Injury causing strain in the spring ligament complex has been shown to result in significant talonavicular laxity. In ankle fractures, when the deep deltoid appears clinically ruptured, SL strain occurs [29]. Therefore, disruption of superficial deltoid fibres may also detension spring ligament fibres to cause dysfunction. SL function is also affected by the integrity of the plantar fascia [23].

In view of the extent of anatomical connections between the various ligamentous bands/complexes, clinical assessments to determine specific failing bands/segments may not be fully functionally isolating [27,30]. For example, the anteromedial drawer test may represent the failure of multiple areas of the DSL, including the tibionavicular band and the deep deltoid layer, without isolating one particular band. Due to the single nature of the DSL complex, failure of one area in this structure may increase the strain on other segments of the DSL complex. For example, tibiospring band laxity may disrupt the superomedial portion of the spring ligament and lead to eventual failure of the whole unit, thereby decreasing stability and function across multiple joints.

Limitations

This study has certain limitations. Firstly, some of the included studies did not indicate the exact number of specimens analysed, which may introduce biases and reduce reproducibility and comparability with other research. Additionally, some researchers used unpaired ankles from different donors without addressing potential differences between them. Consequently, this report does not consider structural variations related to laterality or sex, which could lead to overlooked structural insights.

## Conclusions

The deltoid and spring ligaments can be viewed as a single entity, the DSL complex, with the deep plantar ligament as a distinct structure. This complex is highly specialised, interconnected, and varies based on function and strain. It consists of the deep deltoid and superficial deltospring ligaments, which are linked but anatomically and functionally distinct. The superficial deltospring ligament can be divided into three segments: the anterior segment, which attaches to the navicular; the middle segment, which includes tibia-derived fibres attaching to the sustentaculum tali; and the posterior segment, which consists of fibres posterior to the middle segment. Clinical tests assess segment functions, such as talonavicular abduction for the anterior segment and hindfoot eversion for the middle segment. This study reviews existing ligament terminology and introduces a refined structural classification, emphasising variability and interconnections between the structures.
